# Coagulation Inhibitors in COVID-19 Leading to Compressive Airway Hematoma

**DOI:** 10.7759/cureus.12580

**Published:** 2021-01-08

**Authors:** Amr Mohamed

**Affiliations:** 1 Internal Medicine, Rochester Regional Health, Rochester, USA

**Keywords:** covid 19, covid coagulopathy

## Abstract

We are focusing on three things for every patient in the hospital with COVID-19, namely dexamethasone, remdesivir and enhanced anticoagulation protocols as this had shown improved mortality. However, the bleeding risk in these patients has not been taken into consideration. In our ICU setting at Rochester General hospital, we have seen too many cases with gastrointestinal bleeding and hemoptysis in COVID-19 patients. In this case, we report bleeding related to central access removal related to coagulation inhibitors that lead to airway compression. The aim of this case is to keep bleeding tendency of COVID-19 patients on the radar and to delineate that it has clear severe consequences just as clotting.

## Introduction

It is well known that COVID-19 patients are in a hypercoagulable state. Hence, there is a requirement for specific dosing for blood thinners (even for DVT prophylaxis) in the ICU setting. This is known to improve mortality [1]. However, the bleeding risk in these patients is not taken into consideration. In this case, we describe a fatal bleeding complication from a simple procedure that led to prolonged intubation, all resulting from COVID19 coagulation inhibitors. Hence, we need to see these patients from a wide perspectives as they are at risk for both clotting and bleeding.

## Case presentation

A 72-year-old female with past medical history of hypertension and diabetes was admitted with COVID-19 bronchopneumonia and NSTEMI. Her hospital course was complicated with respiratory failure requiring mechanical ventilation and septic shock requiring norepinephrine. After her shock resolved, the right internal jugular central line was removed and at that time, compressions for about 30 minutes were required to achieve hemostasis. Later, the patient was noticed to have a large hematoma on the right side of the neck causing compression of the airway and prolonging the intubation period to near 25 days.

**Figure 1:**
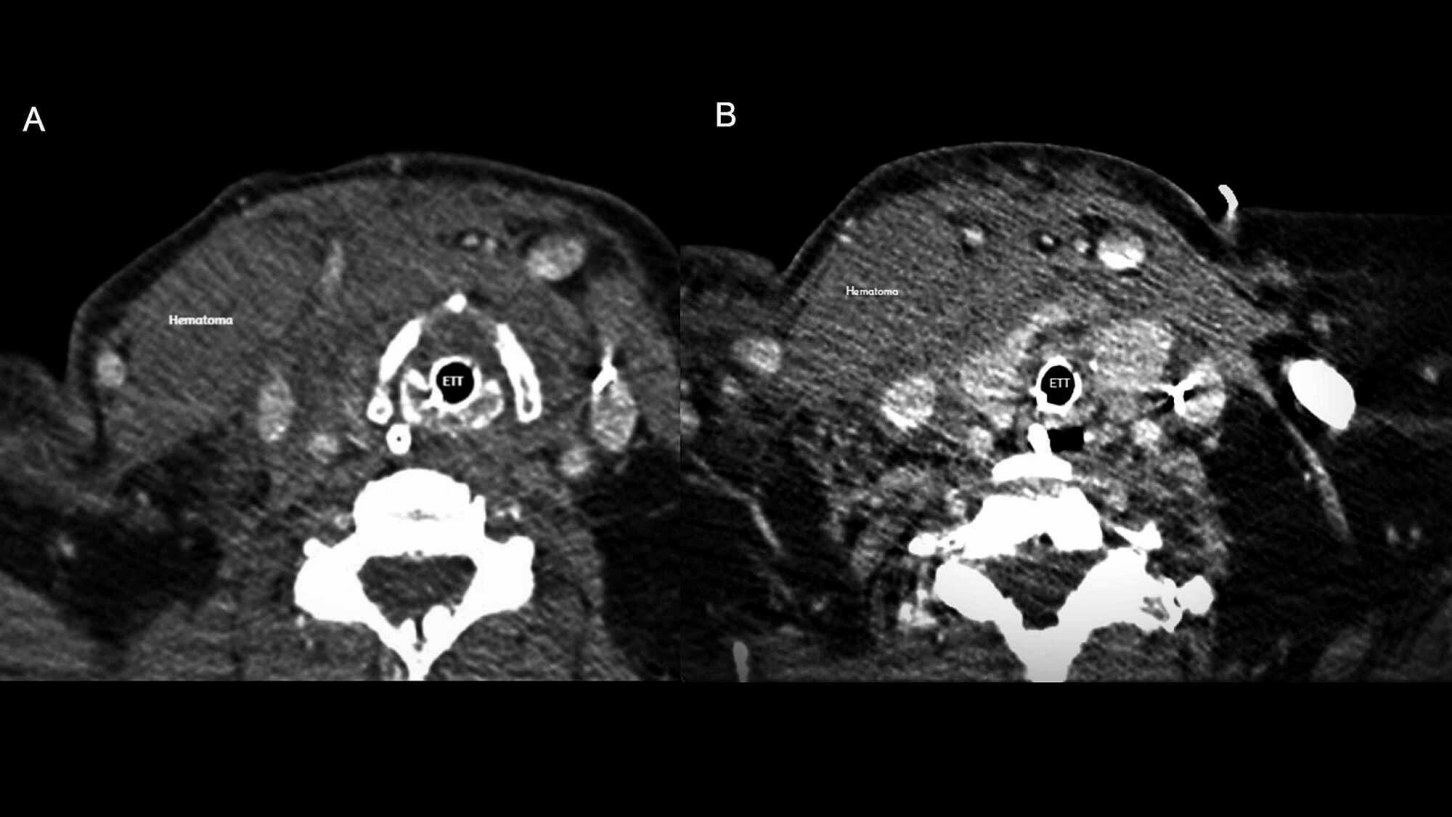
CT scan of the neck with IV contrast showing compressive hematoma of the airway progressing as we go from A to B. ETT stands for endotracheal tube

Significant laboratory values with APTT 85.6 at baseline. A mixing study was done and the APTT just corrected 85.6 to 78.2 (Table 1). Lupus anticoagulant was negative. She had normal coagulation profiles during past admissions and fibrinogen was 450. The picture was not consistent with DIC but rather than with coagulation inhibitor. CT scan of the neck showed a hematoma measuring 11 x 4 cm tracking inferiorly along the chest wall with narrowing and compressing the airway along the endotracheal tube (Figure 1A). This was progressively expanding as shown in sequence B of the CT image (Figure 1B).

**Table 1:** Plasma Mixing study showed failed correction of PT and PTT after mixing

	Ref. Range	
PT	10.2 - 12.9 sec	56.8 (H)
PT IMMEDIATE MIX	10.2 - 12.9 sec	43.5 (H)
PT INCUBATED MIX	10.2 - 12.9 sec	43.1 (H)
APTT	25.1 - 36.5 sec	85.6 (H)
APTT IMMEDIATE MIX	25.1 - 36.5 sec	78.2 (H)
APTT INCUBATED MIX	25.1 - 36.5 sec	78.1 (H)

Initially this case was a dilemma. We first thought about DIC, but the coagulation panel was not consistent with DIC. Subsequently, we considered heparin-induced thrombocytopenia as the platelet count dropped from 250 to 100. However, the platelet factor 4 antibody came back as negative and the thrombocytopenia was attributed to sepsis and COVID-19 induced thrombocytopenia. The results of the mixing study which was consistent with a coagulation inhibitor.

The patient was managed with dexamethasone and remdesivir with conservative management for coagulopathy and we avoided unnecessary vascular lines and unnecessary arterial or venous draws. The coagulopathy later improved with improvement of the underlying COVID-19 infection and her APTT came back to normal.

## Discussion

We are all focused on hypercoagulability while working in the ICU setting for COVID-19 patients as these patients are at very high risk of thrombosis [2]. We usually don’t focus on bleeding risk for these patients unless complication happens or unless it became very apparent like DIC (Figure 1). Other frequently seen causes in our ICU included heparin and COVID-19 induced thrombocytopenia and platelet dysfunction; these are all associated with a poor prognosis [3]. However, there are other causes for coagulopathy that are not very well described in the literature like our case here for coagulation inhibitor. There are few case reports for lupus anticoagulant induced coagulation deficit in COVID-19 patients [4]. However, this was not the scenario in our patient. Thus, we need to be more aware of the bleeding risk in these patients.

In our patient giving fresh frozen plasma to correct the coagulation deficit is unlikely to be helpful as this was due to inhibitors. Thus, we need to be more aware of the cause of coagulopathy as treating the underlying cause is likely to cause the most benefit.

## Conclusions

The main message from this case is that despite COVID-19 patients are in a hypercoagulable state, they are also more liable to bleeding and they may be having other types of coagulation inhibitors other than a lupus anticoagulant.
